# Netrin-1 regulates somatic cell reprogramming and pluripotency maintenance

**DOI:** 10.1038/ncomms8398

**Published:** 2015-07-08

**Authors:** Duygu Ozmadenci, Olivier Féraud, Suzy Markossian, Elsa Kress, Benjamin Ducarouge, Benjamin Gibert, Jian Ge, Isabelle Durand, Nicolas Gadot, Michela Plateroti, Annelise Bennaceur-Griscelli, Jean-Yves Scoazec, Jesus Gil, Hongkui Deng, Agnes Bernet, Patrick Mehlen, Fabrice Lavial

**Affiliations:** 1 Apoptosis, Cancer and Development Laboratory - Equipe labellisée ‘La Ligue', LabEx DEVweCAN, Centre de Recherche en Cancérologie de Lyon, INSERM U1052-CNRS UMR5286, Université de Lyon, Centre Léon Bérard, 69008 Lyon, France; 2 INSERM U935, ESTeam Paris-Sud, Université Paris-Sud, Villejuif, France; 3 Institut de Génomique Fonctionnelle de Lyon, Université de Lyon, Centre National de la Recherche Scientifique (CNRS), Institut National de la Recherche Agronomique, École Normale Supérieure de Lyon, 69364 Lyon, France; 4 CGϕMC UMR 5534 - Université Lyon 1, Campus de la Doua, Bâtiment Gregor Mendel 16, Rue Dubois, 69622 Villeurbanne, France; 5 Center for Life Sciences, Peking University, Yiheyuan Road Haidian District, Beijing 100871, P.R. China; 6 Cytometry Facility, Centre de Cancérologie de Lyon, INSERM U1052-CNRS UMR5286, Université de Lyon, Centre Léon Bérard, 69008 Lyon, France; 7 Anipath, Université de Lyon, Hospices Civils de Lyon, Hôpital Edouard Herriot, Anatomie Pathologique, 69437 Lyon, France; 8 Cell Proliferation Group, MRC Clinical Sciences Centre, Imperial College London, Hammersmith Hospital Campus, Du Cane Road, W12 0NN, London, UK; 9 Cellular Reprogramming and Oncogenesis Laboratory, ATIP/Avenir Laboratory, Centre de Recherche en Cancérologie de Lyon, INSERM U1052-CNRS UMR5286, Université de Lyon, Centre Léon Bérard, 69008 Lyon, France

## Abstract

The generation of induced pluripotent stem (iPS) cells holds great promise in regenerative medicine. The use of the transcription factors Oct4, Sox2, Klf4 and c-Myc for reprogramming is extensively documented, but comparatively little is known about soluble molecules promoting reprogramming. Here we identify the secreted cue Netrin-1 and its receptor DCC, described for their respective survival/death functions in normal and oncogenic contexts, as reprogramming modulators. In various somatic cells, we found that reprogramming is accompanied by a transient transcriptional repression of Netrin-1 mediated by an Mbd3/Mta1/Chd4-containing NuRD complex. Mechanistically, Netrin-1 imbalance induces apoptosis mediated by the receptor DCC in a p53-independent manner. Correction of the Netrin-1/DCC equilibrium constrains apoptosis and improves reprogramming efficiency. Our work also sheds light on Netrin-1's function in protecting embryonic stem cells from apoptosis mediated by its receptor UNC5b, and shows that the treatment with recombinant Netrin-1 improves the generation of mouse and human iPS cells.

## Abstract (web summary)

Reprogramming holds great promise for regenerative medicine but the molecular mechanisms governing the generation of induced pluripotent stem cells remain unclear. Here, the authors reveal functions for the axonal guidance cue Netrin-1 in constraining apoptosis at the early stage of reprogramming and in established pluripotent cells.

## Graphical abstract

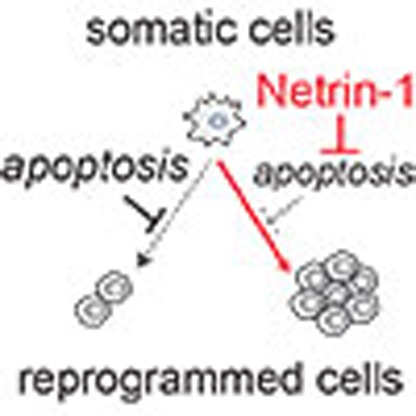

Somatic cell reprogramming to pluripotency involves epigenetic modifications, changes in gene expression, protein degradation and protein synthesis. Reprogramming resets differentiated cells to a pluripotent state and can be achieved by nuclear transfer, cell fusion or transduction of certain transcription factors. The original approach relies on the ectopic expression of the factors Oct4, Sox2, Klf4 and c-myc—collectively known as OSKM^1^,^2^,^3^. The relative flaws in the understanding of the molecular mechanisms governing induced pluripotent stem (iPS) cells generation hinder the efficient derivation of reprogrammed cells without genetic manipulation. Whereas the modulation of the initial OSKM cocktail with other factors has been extensively documented, comparatively little is known about soluble molecules promoting the process, even if such recombinant factors could be highly valuable for therapeutic applications. Several processes acting as reprogramming roadblocks harbour tumour-suppressive activity such as programmed cell death (PCD) and senescence^4^,^5^,^6^. As an example, the p53/Puma axis limits iPS cell generation at the late stage of reprogramming and has been related to the dual pro-oncogenic and pro-PCD effect of c-Myc^7^. However, the mechanisms governing cell death independently of p53/c-Myc in the early days of reprogramming remain unclear^4^,^5^,^6^.

Here we identify the Netrin-1 and its dependence receptor DCC (Deleted in Colorectal Carcinoma) as regulators of somatic cell reprogramming to pluripotency. Netrin-1 is a secreted laminin-related molecule initially identified as an axon guidance cue and more recently proposed as a multifunctional protein implicated both during nervous system development and adult pathologies^8^,^9^. Of interest in the scope of this study, Netrin-1 was shown to act as an oncogene by limiting apoptosis induced by its main dependence receptors DCC and UNC5s (UNC5a, UNC5b, UNC5c and UNC5d)^8^,^9^,^10^,^11^. Using a strategy centred on the genomic regions differentially bound by OSKM in somatic and pluripotent cells, we identify members of the Netrin-1/DCC signalling pathway as putative reprogramming roadblocks. We therefore examine Netrin-1 function during iPS cell generation and reveal that the early stage of the process is associated with a transient Netrin-1 transcriptional repression mediated by Oct4 and Klf4 repressive action on the Netrin-1 promoter. We show that such Netrin-1 deficiency limits reprogramming by engaging DCC-induced apoptosis. In parallel, we demonstrate that Netrin-1 protects established pluripotent cells from apoptosis induced by its receptor UNC5b. Importantly, we demonstrate that the Netrin-1/DCC imbalance is corrected by the non-invasive treatment with recombinant Netrin-1 that improves reprogramming efficiency of human and mouse somatic cells.

## Results

### Netrin-1 level controls iPS cell generation

**Figure 1:**
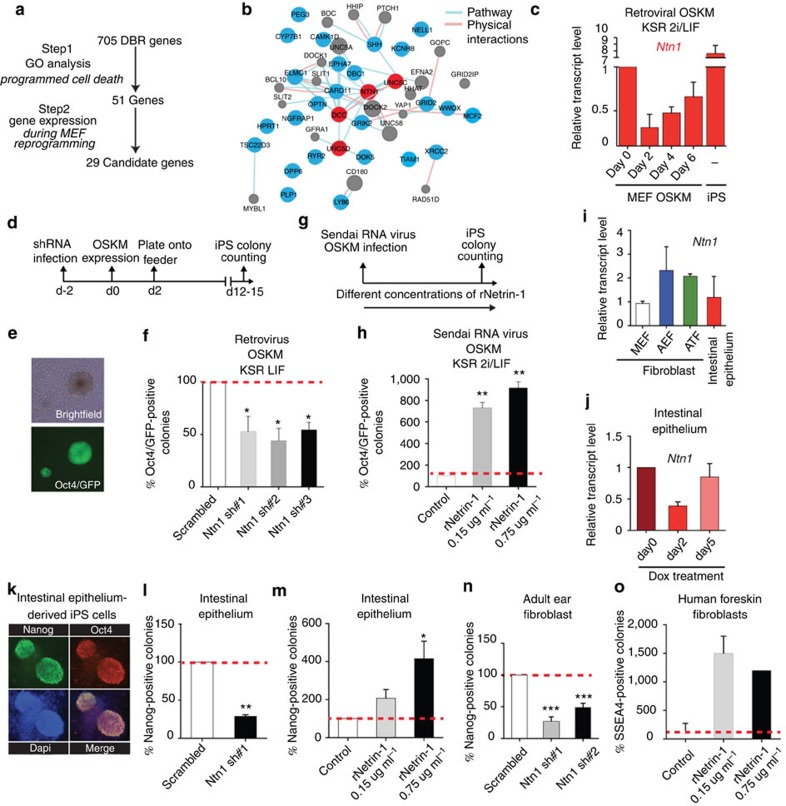
Netrin-1 level governs reprogramming. (**a**) Identification of pluripotent reprogramming impediments. DBR genes were selected using UCSC genome browser ( http://genome.ucsc.edu), gene ontology performed with DAVID ( http://david.abcc.ncifcrf.gov/home.jsp; gene ontology:0008624, 0006917, 0012502, 0043065). Candidate list was refined to 29 genes using RNA-Seq, microarray data and literature. (**b**) Predicted protein interaction network ( http://www.genemania.org). Blue and red dots correspond to candidates, grey dots to potential partners. (**c**) *Ntn1* expression is biphasic during reprogramming. Quantitative reverse transcription–PCR (Q-RT–PCR) depicts *Ntn1* expression levels during reprogramming induced by OSKM retroviral expression. Data are expressed relative to MEF as the mean±s.d. (*n*=3). (**d**) Schematic of the time schedule of reprogramming experiment. (**e**) Typical mouse Oct4/GFP iPS colony 14 days following OSKM infection. Scale bars, 80 μm. (**f**) Netrin-1 depletion reduces reprogramming efficiency. The number of Oct4/GFP-positive colonies produced from sh-scrambled MEFs is set at 100% for each individual experiment. Data are the mean±s.d. (*n*=3). Student's *t*-test, *P*<0.05. (**g**) Schematic of the time schedule of reprogramming experiment. (**h**) Recombinant Netrin-1 (rNetrin-1) effect on mouse Oct4/GFP iPS cells generation induced by OSKM RNA sendai virus. rNetrin-1 (0.15or 0.75 μg ml^−1^) was added daily to the media. Data are the mean±s.d. (*n*=2). Student's *t*-test, *P*<0.01. (**i**) Netrin-1 expression in various somatic cells. Q-RT–PCR depicts *Ntn1* level in mouse embryonic fibroblast (MEF), adult ear (AEF) and tail tip (ATF) fibroblasts and intestinal epithelium. Data are expressed relative to MEF as the mean±s.d. (*n*=3). (**j**) Netrin-1 expression is biphasic during intestinal epithelium reprogramming. Q-RTPCR depicts *Ntn1* expression at day 2 and day 5 post OSKM induction (dox treatment). Data are expressed relative to untreated intestinal epithelium at day 2 and day 5 as the mean±s.d. (*n*=2). (**k**) Oct4/Nanog immunostaining of a miPS colony derived from intestinal epithelium. Scale bars, 100 μm. (**l**) Netrin-1 depletion reduces reprogramming efficiency of intestinal epithelium. The number of Nanog-positive colonies produced from sh-scrambled sample is set at 100%. Data are the mean±s.d. (*n*=2). Student's *t*-test, **P<0.01. (**m**) Recombinant Netrin-1 (rNetrin-1) effect on iPS cells generation from mouse intestinal epithelium. Data are the mean±s.d. (*n*=2). Student's *t*-test, *P<0.05. (**n**) Netrin-1 depeltion reduces reprogramming efficiency of adult ear fibroblasts (AEFs). Nanog immunostaining was performed after 12–14 days of reprogramming. Data are the mean±s.d. (*n*=2). Student's *t*-test, ***P<0.001. (**o**) Recombinant Netrin-1 effect on human iPS colonies emergence from human foreskin fibroblasts. SSEA4-positive hiPS colonies were counted day 26 post infection.

To identify novel pluripotent reprogramming impediments, we developed a method centred on the OSKM ‘differentially bound regions' (DBRs; Fig. 1a)^12^. DBR were identified by comparing the chromatin immunoprecipitation (ChIP)-Sequencing data for OSKM binding in fibroblasts 48 h after OSKM induction and in established pluripotent cells. This approach led to the identification of 264 ‘DBR genomic regions' harbouring a differential OSKM binding in both cell types^12^. We selected the 705 genes located within DBR as candidate genes potentially hindering pluripotent reprogramming (Fig. 1a). We hypothesized that the partial OSKM binding will delay or abrogate their regulation upon reprogramming and might therefore alter iPS cell generation. By performing gene ontology analysis centred on ‘PCD', a major reprogramming impediment, we restricted the list to 51 candidates (Step1). Next, 29 candidates were selected (Step2) due to their dynamic expression during mouse embryonic fibroblasts (MEFs) reprogramming (Supplementary Table 1)^13^. The candidates were subjected to a protein interaction network prediction that revealed an unexpected over-representation of members of the Netrin-1 (Ntn1) signalling pathway with genes corresponding to the Netrin-1 ligand and its receptors DCC, UNC5c and UNC5d (Fig. 1b and Supplementary Table 1).

*Ntn1* expression was assessed during MEF reprogramming induced by OSKM retroviral infection and in the resulting iPS cells grown in 2i/LIF (leukemia inhibitory factor) culture conditions. Such approach revealed a biphasic *Ntn1* expression profile with a marked downregulation during the first 6 days of pluripotent reprogramming followed by a strong reactivation in iPS cells (Fig. 1c). The same pattern, even though with a faster kinetic, is observed in OSKM dox-inducible reprogrammable MEF, ruling out possible side effects of retroviral infection on gene expression (Supplementary Fig. 1a)^14^.

The dynamic expression of Netrin-1 during reprogramming prompted us to investigate whether this factor regulates iPS cell generation. Oct4/GFP knock-in MEF were infected with lentiviral particles encoding a scrambled short hairpin RNA (shRNA) or three different shRNA-targeting Netrin-1 2 days before reprogramming induction by OSKM retroviral infection and 2i/LIF culture conditions (Fig. 1d and Supplementary Fig. 1b). Netrin-1 depletion led to a significant decrease in iPS cell generation efficiency, as denoted by counting GFP-positive colonies activating endogenous Oct4 promoter (Fig. 1e,f). Similar results were obtained by alkaline phosphatase (AP)-positive colony counting, further confirming Netrin-1 effect on iPS cells generation (Supplementary Fig. 1c,d).

### Recombinant Netrin-1 improves reprogramming efficiency

Conversely, Netrin-1 downregulation at the early stage of reprogramming prompted us to ask whether the non-invasive treatment with soluble recombinant Netrin-1 (rNetrin-1) could compensate for such endogenous deficiency and improve reprogramming features. We induced Oct4/GFP MEF to reprogramme using non-integrative RNA Sendai virus and 2i/LIF culture conditions. In such settings, the treatment with rNetrin-1 led to a eightfold increase in the number of Oct4/GFP-positive colonies (Fig. 1g,h). A similar trend was observed with other reprogramming strategies such as OSKM retroviral infection (Supplementary Fig. 1e–g). Conversely, and as expected, a Netrin-1-blocking antibody reduces AP+ colony formation (Supplementary Fig. 1h,i).

We then asked whether Netrin-1 effect on iPS cell generation was restricted to MEFs or could be broadened to other adult somatic cells. We derived various primary cells and detected comparable Netrin-1 expression levels in adult fibroblasts from ear (AEF) or tail tip (ATF) and in intestinal epithelium where Netrin-1 is involved in cell survival and homeostasis (Fig. 1i)^10^. Interestingly, OSKM expression induced by doxycycline (dox) treatment in cultured intestinal epithelium led to the same transient *Ntn1* downregulation than in MEF (Fig. 1j), whereas such phenomenon was not observed in AEF (Supplementary Fig. 1j). Next, we plated intestinal epithelium on irradiated feeder and iPS cells formation by dox treatment. As reported, AP-positive iPS colonies reactivating the naive pluripotency marker Nanog emerged around day 15 (Fig. 1k and Supplementary Fig. 1k)^14^. In such context, we demonstrated that Netrin-1 knockdown, before reprogramming induction, reduced by 75% the number of Nanog-expressing iPS colonies, whereas recombinant Netrin-1 (rNetrin-1) treatment increased iPS cells generation by a fourfold factor (Fig. 1l,m). Netrin-1 depletion also reduced iPS cell generation efficiency from AEF (Fig. 1n).

We next assessed whether recombinant Netrin-1 treatment improves human somatic cells reprogramming. Treatment of human foreskin fibroblasts (HFFs) with rNetrin-1 induced to reprogramme by non-integrative RNA Sendai virus led to a 15-fold increase in reprogramming efficiency, as evaluated by counting the number of SSEA4-positive colonies (Fig. 1o). These findings identify (i) Netrin-1 as a novel factor governing iPS cell formation and (ii) recombinant Netrin-1 as a novel soluble factor improving reprogramming efficiency of various human and mouse somatic cells.

**Figure 2:**
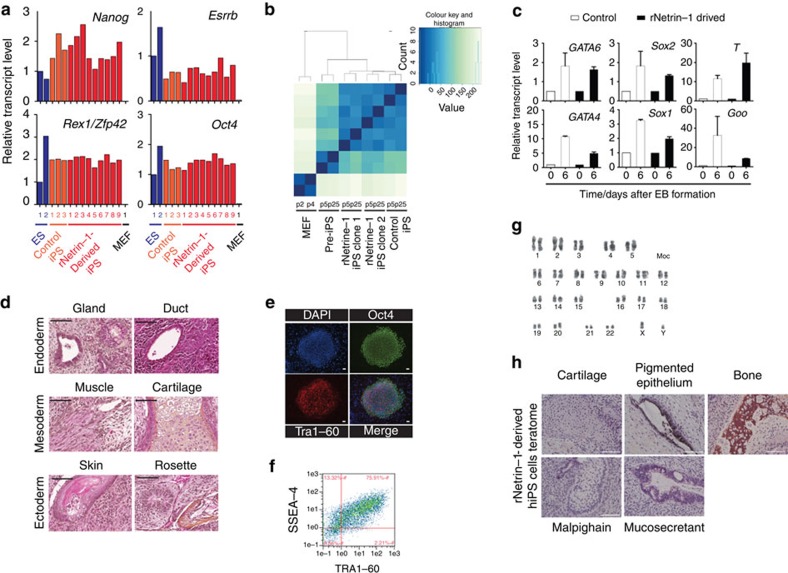
Recombinant Netrin-1 treatment is not detrimental to iPS cells quality. (**a**) Naive pluripotency markers expression in ‘control' and ‘rNetrin-1 derived' miPS cells from RNA Sendai virus. Mouse ES cells, three ‘control' iPS clones, nine ‘rNetrin-1-derived' iPS clones and MEF were analysed for *Nanog*, *Esrrb, Rex1/Zfp42* and *Oct4* expression by quantitative reverse transcription–PCR. (**b**) Heatmap of the euclidean distances between the samples as calculated from the variance stabilizing transformation of the count data (DESeq). Samples consist of Oct4/GFP MEF, mouse pre-iPS, ‘control' and ‘rNetrin-1-derived' iPS clones derived by OSKM retroviral infection. (**c**) ‘Control' and ‘rNetrin-1-derived' mouse iPS cells form embryoid bodies containing derivatives of the three germ layers. Data are normalized to housekeeping genes and expressed relative to t0 as the mean±s.d. (*n*=3). (**d**) Teratoma histological analysis from ‘ rNetrin-1-derived' miPS cells showing contribution to the three germ layers. Scale bars, 100 μm. (**e**) Pluripotency markers expression in ‘rNetrin-1-derived' human iPS cells. Immunostainings for OCT4 and Tra1-60 were performed on a hiPS colony. Scale bars, 50 μm. (**f**) SSEA4 and TRA1-60 FACS analysis of ‘rNetrin-1-derived' hiPS cells. (**g**) Karyotype of ‘rNetrin-1-derived' hiPS cells. (**h**) Histological analysis of a ‘rNetrin-1-derived' hiPS cells teratoma. Scale bars, 200 μm.

In a therapeutic perspective of improving reprogramming efficiency, we addressed whether recombinant Netrin-1 (rNetrin-1) treatment is detrimental to the iPS cell quality. We characterized pluripotency features and genomic stability of mouse (miPs) and human iPS (hiPS) cells derived with rNetrin-1. We expanded mouse Oct4/GFP iPS (miPS) clones obtained with integrative and non-integrative methods in standard conditions (‘control') or with rNetrin-1 (0.6 μg ml^−1^; ‘rNetrin-1 derived'). The 9 ‘rNetrin-1 derived' miPS clones generated using RNA sendai virus harboured similar levels for *Nanog*, *Rex1* and *Esrrb* RNA than the three ‘control' miPS clones and mouse embryonic stem (ES) cells, showing that rNetrin-1 is not detrimental to the transcriptional reactivation of such naive pluripotent circuitry (Fig. 2a). In parallel, we performed RNA-sequencing analysis to compare Oct4/GFP MEF, partially reprogrammed (pre-) miPS cells, ‘control' miPS cells and two different ‘rNetrin-1 derived' miPS clones generated by OSKM retroviral infection (Fig. 2b and Supplementary Fig. 2a). The mouse pre-iPS cells do not reactivate the Oct4/GFP endogenous locus and do not silence the retroviral transgenes^15^. The calculation of the euclidian distances revealed a strong correlation between the ‘control' and the two ‘rNetrin-1-derived' miPS clones, confirming similar transcriptome resetting in both conditions (Fig. 2b). In particular, ‘rNetrin-1-derived' miPS cells harbour similar *Nanog*, *Esrrb* and *Dppa3* levels than ‘control' miPS cells, whereas the pre-iPS cells failed at reactivating these genes (Supplementary Fig. 2b). After 40 passages in culture, the quantification of major chromosomal alterations did not reveal any significant difference between miPS cell lines, suggesting that rNetrin-1 treatment does not impact massively chromosomal stability (Supplementary Fig. 2c). In terms of differentiation potential, we showed that ‘rNetrin-1-derived' miPS cells turn off the endogenous Oct4/GFP promoter activity following LIF withdrawal for 5 days (Supplementary Fig. 2d). Embryoid body formation assays demonstrated their ability to give rise to derivatives of the three germ layers (Fig. 2c). The histological analysis of teratoma formed by ‘rNetrin-1 derived' miPS cells confirmed the presence of tissues representing all three germ layers (Fig. 2d). In parallel, two hiPS cell lines derived from foreskin fibroblasts with rNetrin-1 were characterized at the molecular and functional levels. As shown in Fig. 2e,f, ‘rNetrin-1 derived' hiPS cells express the pluripotency-associated markers OCT4, TRA1-60 and SSEA4. Karyotype analysis revealed no abnormalities and teratoma formation confirmed the presence of derivatives of the three germ layers (Fig. 2g,h). Altogether, these results suggest that the treatment with recombinant Netrin-1 is not detrimental to mouse and hiPS cell features.

### Netrin-1 limits DCC-induced apoptosis during reprogramming

**Figure 3:**
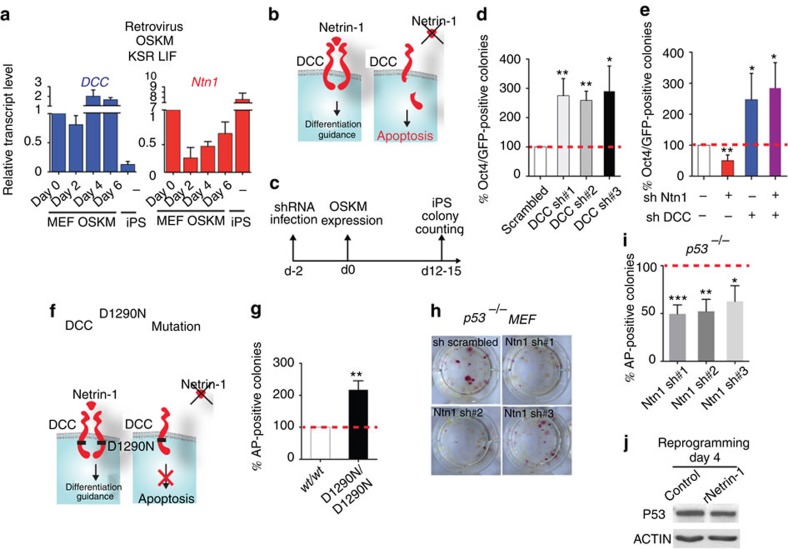
DCC pro-apoptotic activity limits reprogramming occurrence. (**a**) DCC expression is maintained at the early stage of reprogramming. Quantitative reverse transcription–PCR depicts *DCC* and *Ntn1* expression profile during reprogramming. Data are expressed relative to MEF as the mean±s.d. (*n*=3). (**b**) Schematic depicting the dependence receptor paradigm. In presence of its ligand Netrin-1, DCC transduces a positive signalling leading to differentiation and guidance cues. In the absence of or in Netrin-1-limiting conditions, DCC actively induces caspase-dependent apoptosis. (**c**) Schematic of the time schedule of reprogramming experiment. (**d**) DCC depletion improves reprogramming efficiency. The number of Oct4/GFP colonies produced from sh-scrambled MEFs is set at 100% for each individual experiment. Data are the mean±s.d. (*n*=3). Student's *t*-test, *P<0.05 or **P<0.01. (**e**) Netrin-1 depletion effect is rescued by DCC knockdown. Data are the mean±s.d. (*n*=3). Student's *t*-test, *P<0.05 or **P<0.01. (**f**) Scheme depicting DCC^D1290N^ point mutant model. In the presence of Netrin-1, DCC-positive signalling remains unaffected. In settings of ligand absence, DCC pro-apoptotic activity is abrogated. (**g**) DCC pro-apoptotic activity abrogation improves reprogramming efficiency. MEFs were derived from embryos of *DCC*^*wt/D1290N*^ mice intercrosses. *DCC*^*wt/wt*^ and *DCC*^*D1290N/D1290N*^ were subjected to reprogramming and AP-positive colonies scored at days 12–14. The number of colonies from *MEF*^*wt/wt*^ is set at 100% for each individual experiment. Data are the mean±s.d. (*n*=3). Comparable transduction efficiencies were achieved with *DCC*^*wt/wt*^ and *DCC*^*D1290N/D1290N*^ MEF. Student's *t*-test, *P<0.05. (**h**–**j**) Netrin-1 effect is mainly p53 independent. (**h**–**i**) Netrin-1 depletion reduces reprogramming efficiency of *p53*^*−/−*^ MEF. Data are the mean±s.d. (*n*=3). Student's *t*-test, *P<0.05 or **P<0.01 or ***P<0.001. (**j**) p53 protein levels are not modulated by rNetrin-1 treatment. Western blot for p53 and ACTIN was performed on MEF treated with dox for 4 days in the presence or absence of recombinant Netrin-1 (0.75 μg ml^−1^).

To decipher the mechanisms downstream of Netrin-1, the expression of its receptors was assessed during MEF reprogramming. In the first 6 days, *DCC* expression remains steady, whereas *UNC5b* and *UNC5c* are rapidly downregulated (Fig. 3a and Supplementary Fig. 2e). DCC belongs to the functional family of dependence receptors sharing the ability of triggering apoptosis in the absence of their ligand (Fig. 3b). The expression of such receptor thus renders the cell dependent for its survival on ligand availability^8^,^16^,^17^. In Netrin-1-limiting conditions, unbound DCC triggers apoptosis via a cleavage of its intracellular domain^18^. We asked whether Netrin-1 transient downregulation limits reprogramming by engaging DCC-induced apoptosis. To test this hypothesis, DCC expression was silenced with shRNA lentiviral particles before MEF reprogramming induced by OSKM retroviral infection. DCC knockdown using three different shRNA significantly improves reprogramming efficiency, as denoted by counting the number of Oct4/GFP colonies (Fig. 3c,d and Supplementary Fig. 2f). More importantly, the Netrin-1 depletion effect is rescued by DCC knockdown, identifying the pair Netrin-1/DCC as a novel mediator of reprogramming (Fig. 3e). In contrast, silencing of the Netrin-1 receptor UNC5b did not modify iPS cell generation efficiency (Supplementary Fig. 2g,h). We generated a mice model in which a DCC point mutation at the critical amino acid required for intracellular cleavage (aspartic acid 1290) is sufficient to block DCC-induced apoptosis while leaving unaffected the positive DCC signalling observed upon Netrin-1 binding (Fig. 3f)^19^. MEFs derived from *DCC*^*wt/wt*^ and *DCC*^*D1290N/D1290N*^ littermate embryos were reprogrammed by retroviral OSKM infection and 2i/LIF culture conditions. *DCC*^*D1290N/D1290N*^ MEFs gave rise to iPS cell colonies with a slightly higher efficiency than *DCC*^*wt/wt*^ MEFs, indicating that DCC effect on reprogramming efficiency is at least partly due to its pro-apoptotic activity (Fig. 3g). Recombinant Netrin-1 (rNetrin-1) effect was compared during *DCC*^*wt/wt*^ and *DCC*^*D1290N/D1290N*^ MEF reprogramming. The rNetrin-1 and Netrin-1 blocking antibody effects are nearly abrogated in *DCC*^*D1290N/D1290N*^ MEF, confirming that Netrin-1 effect is partly mediated by the inhibition of DCC pro-apoptotic activity (Supplementary Fig. 2i,j).

Reprogramming factors, especially c-Myc, activate the p53 pathway whose alleviation reduces apoptosis and improves iPS cells generation^4^,^5^,^6^. We investigated whether Netrin-1 effect was dependent on c-Myc/p53. First, reprogramming experiments were performed without c-Myc (OSK) using integrative and non-integrative methods^4^. We demonstrated that, in the absence of c-Myc, the modulation of Netrin-1 level still impacts iPS cell generation, even if at a lesser extent (Supplementary Fig. 2k,l). To assess whether Netrin-1 effect was p53 mediated, *p53*^*−/−*^ MEFs were infected with shRNA against *Ntn1* before OSKM reprogramming. Such approach still led to a significant decrease in the number of AP-positive colonies (Fig. 3h,i). The treatments with rNetrin-1 or Netrin-1 blocking antibody are also still effective in the absence of p53 (Supplementary Fig. 2m,n). Moreover, p53 protein levels remained unchanged by rNetrin-1 treatment at reprogramming day 4 (Fig. 3j). Taken together, these data support the view that DCC pro-apoptotic activity limits pluripotent reprogramming mainly in a c-Myc/p53-independent manner.

**Figure 4:**
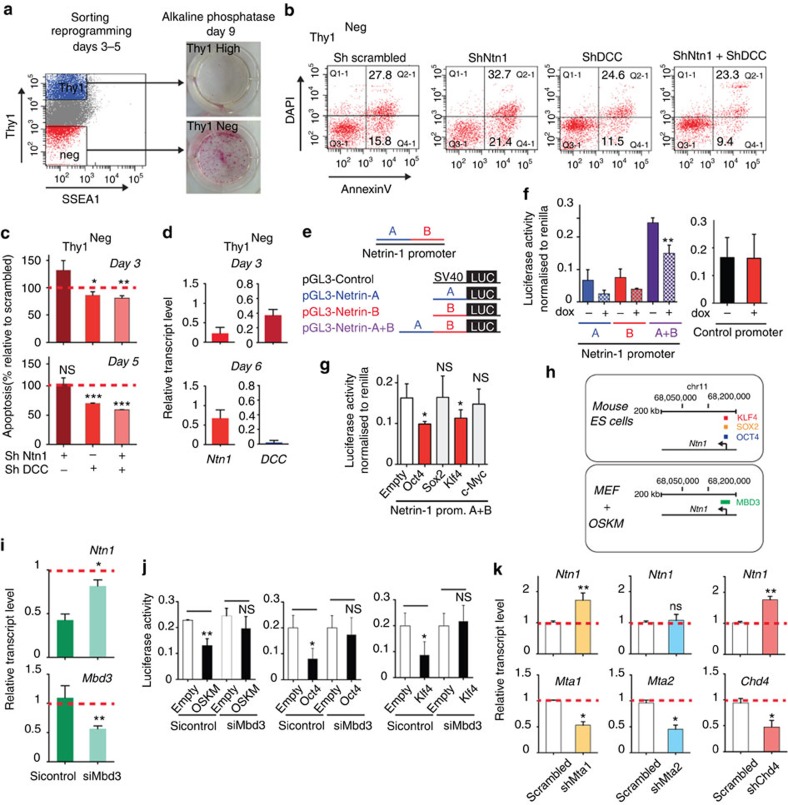
Ntn1/DCC balance modulates apoptosis at the early stage of reprogramming. (**a**) Representative FACS profile of thy1^High^ and thy1^Neg^ cells 3–5 days post OSKM infection. Cells were sorted and plated back in reprogramming media for 6–9 additional days before AP activity staining. (**b**) Representative FACS profile of apoptosis in thy1^Neg^ cells following Ntn1 and/or DCC depletion. Apoptotic cells percentages are indicated. (**c**) Quantification of thy1^Neg^ apoptotic cells following Ntn1 and/or DCC depletion at days 3 and 5. The apoptotic cell percentage in sh-scrambled thy1^Neg^ MEFs is set at 100%. Data are the mean±s.d. (*n*=3). Student's *t*-test, *P<0.05 or **P<0.01 or ***P<0.001; NS, non-significant. (**d**) *Ntn1* and *DCC* expression levels in thy1^Neg^ cells. Cells were sorted at days 3 and 6 post OSKM retroviral infection. Quantitative reverse transcription–PCR (Q-RT–PCR) data are expressed relative to MEF as the mean±s.d. (*n*=3). (**e**) Scheme depicting Netrin-1 promoter constructs: part A, B and A+B^23^. pGL3-control vector with SV40 promoter and enhancer driving luciferase expression is used as ‘control vector'. (**f**) Dox-inducible MEFs were transfected with Netrin-1 promoter luciferase constructs and treated with or without dox for 48 h. Data are normalized to Renilla activity and expressed as the mean±s.d. (*n*=2). Student's *t*-test, *P*<0.05. (**g**) Regulation of Netrin-1 promoter activity by single reprogramming factors. HEK293T cells were transfected with Netrin-1 promoter part A+B and reprogramming factors. Data are the mean±s.d. (*n*=3). Student's *t*-test, *P*<0.05. (**h**) Mbd3 is recruited to Ntn1 promoter upon reprogramming. ChIP-seq resource was employed to assess Ntn1 promoter occupancy in mouse ES cells and in MEF following OSKM induction. Data are extracted from refs 22, 26. Mbd3 is recruited on chromosome 11 (position 68209776-68210746 NCBI36/mm8) in the vicinity of Oct4- (chr11 68210308-68210332), Sox2- (chr11 68210051-68210500) and Klf4- (chr11 68210297-68210303) binding sites. (**i**) Netrin-1 transcriptional silencing is rescued by Mbd3 depletion. MEFs were transfected with Mbd3 siRNA 24 h before OSKM infection and cells harvested 36 h post OSKM infection. Data are the mean±s.d. (*n*=3). Student's *t*-test, *P<0.05 or **P<0.01. (**j**) OSKM repressive effect on Ntn1 promoter activity is mediated by Mbd3. HEK293T cells were transfected with Mbd3 siRNA before Ntn1 promoter luciferase construct and reprogramming factors. Luciferase activity was assessed 48 h post transfection and normalized to Renilla activity. Data are the mean±s.d. (*n*=2). Student's *t*-test, *P<0.05. (**k**) Netrin-1 silencing is mediated by Mta1 and Chd4. MEFs were infected with lentiviral particles targeting the NuRD complex members Mta1, Mta2 and Chd4 and reprogramming induced 48 h later. Q-RTPCR depicts *Ntn1*, *Mta1*, *Mta2* and *Chd4* expression levels at reprogramming day 2. Data are the mean±s.d. (*n*=3). Student's *t*-test, *P<0.05 or **P<0.01.

We next analysed apoptosis occurrence at the early stage of reprogramming by FACS combining the cell surface markers thy1 and SSEA1 with 4,6-diamidino-2-phenylindole (DAPI) and Annexin V^15^,^20^,^21^. The starting MEF population express thy1, whereas SSEA1 is repressed (thy1^Positive^/SSEA1^Negative^). FACS analysis performed at days 3 and 5 post OSKM infection confirmed the emergence of a thy1^Negative^/SSEA1^Negative^ subpopulation (thy1^Neg^) enriched in reprogramming cells, as shown by morphology and AP staining (Fig. 4a and Supplementary Fig. 3a). We showed that, in the early days of reprogramming, Netrin-1/DCC ratio modulation directly impacts apoptosis occurrence with thy1^Neg^ cells being particularly sensitive to such phenomenon (Fig. 4b,c and Supplementary Fig. 3b–e). Following Ntn1 silencing, the percentage of thy1^Neg^ apoptotic cells is increased by 30% at reprogramming day 3 (Fig. 4b,c and Supplementary Fig. 3e). Conversely, following DCC knockdown, the percentage of thy1^Neg^ apoptotic cells is decreased by 20% and 40% at reprogramming day3 and 5, respectively. As expected, Ntn1 and DCC double knockdown led to a reduction in apoptosis occurrence (Fig. 4b,c and Supplementary Fig. 3e). Taken together, these data support the view that the early Netrin-1/DCC imbalance limits iPS generation by inducing DCC-mediated apoptosis predominantly in thy1^Neg^ reprogramming cells. *Ntn1* and *DCC* expression levels were next assessed at this stage of reprogramming. At day 3, *Ntn1* expression is strongly downregulated in thy1^Neg^ cells, whereas *DCC* remains steady—situation favouring DCC-mediated cell death (Fig. 4d). However, 3 days later, the pattern is inverted with *Ntn1* reactivation and *DCC* silencing, possibly reflecting the elimination of the DCC-expressing cells (Fig. 4d). The *Ntn1* activation is maintained at day 9 in the thy1^Negative^/SSEA1^Positive^ cells co-expressing Nanog and in established iPS cells (Supplementary Fig. 3f)^15^,^21^.

### Oct4, Klf4 and the NuRD complex repress Netrin-1 expression

We then asked which epigenetic mechanism triggers Netrin-1 transient transcriptional repression. In agreement with its location within DBR, the Ntn1 locus is occupied by Oct4, Sox2 and Klf4 in mouse ES cells (mm8 genome browser: Oct4-binding chr11: 68210308-68210332, Sox2-binding chr11: 68210051-68210500, Klf4-binding chr11: 68210297-68210303; Supplementary Fig. 4a)^12^,^22^. We evaluated OSKM effect on the activity of Netrin-1 promoter^23^. Luciferase assays performed on OSKM dox-inducible MEF transfected with different promoter constructs demonstrated the ability of the reprogramming cocktail to repress specifically Netrin-1 promoter activity, whereas control SV40 promoter activity remains steady (Fig. 4e,f). A similar approach was employed in HEK293T cells with single reprogramming factors to reveal that Oct4 and Klf4 are responsible for Netrin-1 promoter repression (Fig. 4g).

Different epigenetic complexes, such as Polycomb, WDR5 and NuRD, are associated with early development and connected to the reprogramming factors activity^24^,^25^,^26^,^27^. Among them, the nucleosome remodelling and deacetylase (NuRD) complex member Mbd3 has been identified as a strong reprogramming roadblock, with OSKM inducing a global redistribution of Mbd3 on target genes in MEF (1,177 binding regions in MEF compared with 8,657 after OSKM induction)^26^. ChIP-Sequencing data revealed that, upon dox induction in MEF, Mbd3 gets specifically recruited on the Netrin-1 promoter, at a genomic location that corresponds exactly to Oct4-, Sox2- and Klf4-binding site in mouse ES cells (mm8 genome browser: Mbd3-binding chr11: 68209776-68210746; Fig. 4h)^12^. As depicted in Fig. 4i, Mbd3 knockdown before reprogramming induction led to a rescue of Ntn1 downregulation. In agreement with this finding, comparison of Netrin-1 expression profile during MEF^wt/wt^ and Mbd3^fl/-^ reprogramming shed light on a significantly higher induction of *Ntn1* in day 8 Mbd3^fl/-^ MEF reprogramming than in day 11 MEF^wt/wt^, whereas *Ntn3* expression profile remains unaffected (Supplementary Fig. 4b). Luciferase assays confirmed that Oct4 and Klf4 repressive effect on Netrin-1 promoter activity is mainly mediated by Mbd3 (Fig. 4j). Moreover, depletion of the other NuRD components Mta1 and Chd4 before reprogramming also rescued *Ntn1* downregulation, whereas Mta2 knockdown had no effect (Fig. 4k). Taken together, these data demonstrate that a Mbd3/Mta1/Chd4-containing NuRD complex is responsible for the transient Netrin-1 transcriptional repression observed upon reprogramming.

### Netrin-1 limits apoptosis in pluripotent cells

**Figure 5:**
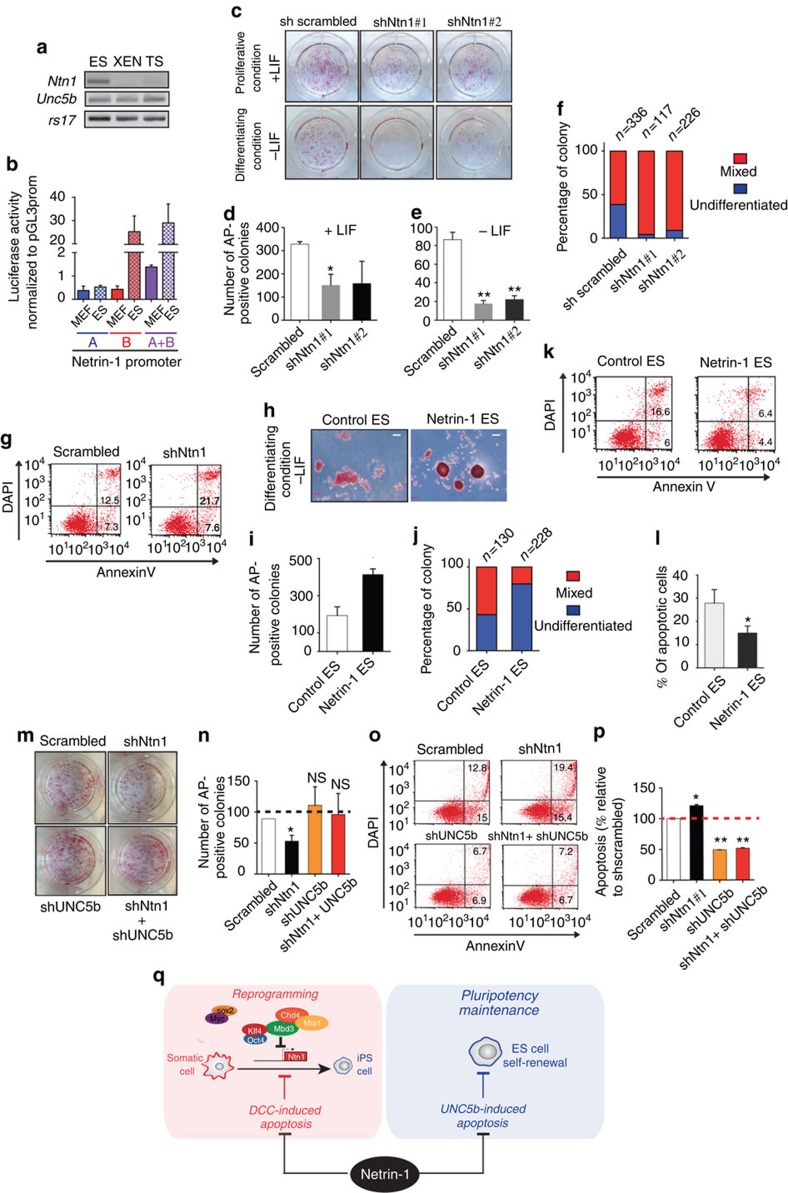
Netrin-1 promotes pluripotency features *in vitro*. (**a**) *Netrin-1* and *UNC5b* expression in blastocyst-derived stem cells. Semi-quantitative RTPCR depicts *Ntn1*, *UNC5b* and housekeeping *rs17* expression levels. (**b**) Netrin-1 promoter activity in pluripotent versus somatic contexts. Netrin-1 promoter constructs were transfected in mouse embryonic stem (mES) and mouse embryonic fibroblasts (MEF) cells. Data are normalized to Renilla activity and expressed as the mean±s.d. (*n*=3). Student's *t*-test, *P*<0.05. (**c**–f) Netrin-1 knockdown decreases colony formation ability of mES cells. sh scrambled, shNtn1#1 and shNtn1#2 mES cells were plated at low density and grown for 6 days in the presence (proliferating condition) or absence (differentiation condition) of LIF before AP staining. (**c**) Colony formation assay results. (**d**,**e**) Counting of AP-positive colonies obtained in the presence (**d**) or in the absence (**e**) of LIF. Data are expressed as the mean±s.d. (*n*=3). Student's *t*-test, *P*<0.05. (**f**) Netrin-1 depletion affects mES cells pluripotency. ‘Unidifferentiated' and ‘mixed' colonies were scored after colony formation assay. The *n* indicates the number of colonies counted. Data are representative of three independent experiments. (**g**) Representative FACS profile of apoptosis in sh scrambled and shNtn1#1 mES cells. Cells were plated at low density for 3–5 days and stained with Annexin V/DAPI. (**h**–**j**) Netrin-1 exogenous expression improves mES cells self-renewal properties. (**h**) AP pictures of ‘control' and ‘Netrin-1-expressing' mES cells. Scale bars, 100 μm. (**i**) Counting of AP-positive colonies. Same protocol as **c**. Data are expressed as the mean±s.d. (*n*=3). (**j**) ‘Undifferentiated' and ‘mixed' colonies were scored after colony formation assay. Data are representative of three independent experiments. (**k**) Representative FACS profile of apoptosis in ‘control' and ‘Netrin-1-expressing' mES cells. (**l**) Quantification of apoptosis in ‘control' and ‘Netrin-1-expressing' ES cells. Data are the mean±s.d. (*n*=3). Student's *t*-test, *P<0.05. (**m**–**p**) Netrin-1 depletion effect is rescued by UNC5b knockdown. (**m**) Bright-field pictures of colony formation assay results. (**n**) AP-positive colony counting. Data are expressed as the mean±s.d. (*n*=3). (**o**,**p**) Netrin-1 depletion effect on apoptosis is rescued by UNC5b knockdown. (**o**) Representative FACS profile of apoptosis in sh scrambled, shNtn1, shUNC5b and shNtn1+shUNC5b mES cells. (**p**) Quantification of apoptosis following Netrin-1 and/or UNC5b depletion. The apoptotic cells percentage in sh-scrambled mES cells is set at 100%. Data are the mean±s.d. (*n*=2). Student's *t*-test, *P<0.05 or **P<0.01. (**q**) Model recapitulating Netrin-1 function in reprogramming and pluripotency maintenance.

Finally, owing to the high Netrin-1 expression level in iPS cells, we asked whether Netrin-1 contributes to pluripotency maintenance. Taking advantage of blastocyst-derived stem cells *in vitro*, we showed that *Ntn1* expression is confined to ES cells and absent from the extra-embryonic-derived TS and XEN cells, whereas *UNC5b* is detected in the three *in vitro* cellular models, even if at a reduced level compared with MEF (Fig. 5a). We next showed that the Netrin-1 promoter activity is strong in pluripotent ES cells compared with MEF, because of genomic elements located in the promoter part B (Fig. 5b). We asked whether Netrin-1 is expressed during pre-implantation development but *Ntn1* mRNA could not be detected in blastocysts by *in situ* hybridization. This observation was confirmed by exploiting single-cell PCR resource performed in mouse blastomeres and in ES cells^28^. *Ntn1* and its receptors (*DCC*, *UNC5a-d)* are not detected at the blastocyst or epiblast stages *in vivo* but *Ntn1* and *UNC5b* are activated during the ES cell derivation process (Supplementary Fig. 4c). To evaluate Netrin-1 function, Netrin-1 knockdown ES cells were established by lentiviral infection with two different shRNA (Fig. 5c–g and Supplementary Fig. 4d). In a colony formation assay, we showed that Netrin-1 depletion led to a drastic reduction in the number of AP-positive colonies obtained in the presence or absence of LIF, demonstrating that Netrin-1 contributes to maintain ES cell self-renewal properties (Fig. 5c–e). Moreover, when the morphology of the mouse ES colonies was compared, a 30% decrease in the percentage of ‘undifferentiated' colonies (round, strongly and homogeneously positive for AP) and a concomitant 30% increase in the percentage of ‘mixed' colonies (weak and heterogeneous AP staining) was detected with the Netrin-1-depleted population, suggesting a defect in the stability of the pluripotent state (Fig. 5f). This phenotype could be due to a defect in cell cycle and/or survival in such challenging conditions. No significant change on cell cycle could be detected upon Netrin-1 knockdown (Supplementary Fig. 4e). However, Netrin-1 depletion led to a significant increase of the percentage of apoptotic cells, whereas necrotic occurrence remains unaffected, demonstrating a protective effect of Netrin-1 in pluripotent cells (Fig. 5g and Supplementary Fig. 4f). Conversely, stable mouse ES clones expressing exogenously Netrin-1 present an improved self-renewal ability in colony formation assay (Fig. 5h–j). The effect on the ES cell morphology was particularly striking with colonies that remained undifferentiated even after 5 days in differentiation conditions in the absence of LIF (Fig. 5h). Interestingly, such Netrin-1 exogenous expression reduced significantly apoptosis occurrence in ES cells (Fig. 5k,l and Supplementary Fig. 4g). Owing to *UNC5b* expression in ES cells, we asked whether Netrin-1 function was mediated by this dependence receptor. UNC5b knockdown rescues Netrin-1 depletion effect on colony formation assay and apoptosis occurrence, identifying the pair Netrin-1/UNC5b as novel regulators of apoptosis in pluripotent ES cells *in vitro* (Fig. 5m–p and Supplementary Fig. 4h).

## Discussion

Together, the data presented here described the importance of the dependence receptor paradigm both in the regulation of reprogramming and of pluripotency, the balance between Netrin-1 and its receptors DCC or UNC5b being key to regulate both processes. Dependence receptors form a family of more than 20 membrane receptors that are not linked by their structure, but by the common functional trait of triggering active apoptosis in the absence of their respective ligand. Thus, cells expressing these types of receptor are dependent on the presence of ligands in the extracellular environment to survive.

Along this line, it was recently formerly demonstrated that the Netrin-1/DCC pair plays a major role in cancer with DCC constraining tumour progression^19^,^29^ and with Netrin-1-promoting tumour progression^10^,^30^ via their respective pro-apoptotic/survival activity. This dual activity may also be implicated in neuronal navigation and developmental angiogenesis^11^,^31^

In this study, we identified unexpected functions for the secreted cue Netrin-1 in regulating iPS cell generation and pluripotency maintenance (Fig. 5q)^26^. On the one hand, we propose an integrated model deciphering how the early dichotomous function of OSKM leads to the recruitment of a repressive Mbd3/Mta1/Chd4-containing NuRD complex to the Ntn1 promoter, leading to Netrin-1 deficiency. This finding reinforces and broadens the view that early OSKM impaired binding to the somatic genome, combined with the recruitment of antagonistic epigenetic complexes, result in aberrant gene expression that is detrimental for iPS cell generation^12^,^26^. We show that the destabilization of the balance between Netrin-1 and its receptor DCC leads to apoptosis induction at the early stage of reprogramming and reduces iPS cell generation. Importantly, we established Netrin-1 as a recombinant protein able to improve the generation of miPS and hiPS cells under specific culture conditions. On the other hand, our work shed light on another Netrin-1 function in ES cell self-renewal, notably by constraining UNC5b-mediated apoptosis. Our work, by providing novel insights into the early stage of reprogramming and pluripotency maintenance, should shorten the road to develop clinically useful iPS cells.

## Methods

### Cell culture and RNAi experiments

MEFs were derived from E13.5 embryos from different backgrounds. Mouse pre-iPS, iPS and Cgr8 ES cells were cultured on gelatin or on irradiated MEF in KSR+LIF or KSR 2i+LIF^32^ as previously described^33^. Mouse XEN and TS cells were grown as previously reported^33^. Intestinal epithelium was grown and induced to form iPS cells, as previously reported^14^. 293T and plat-E cells were grown in DMEM supplemented with 10% FCS and penicillin/streptomycin. For reprogramming, when stipulated, 2i components were added 5–6 days post OKM induction (infection or dox treatment). PD0325901 and chiron were purchased from Merck Millipore. shRNA experiments were performed using pLKO.1 vectors and short interfering RNA (siRNA) using esirna from Sigma (SHCLNG-NM_008744 for Ntn1, SHCLNG-NM_007831 for DCC, SHCLNG-NM_029770 for UNC5b and EMU022741 for Mbd3, SHCLNG-NM_054081 for Mta1, SHCLNG-NM_11842 for Mta2 and SHCLNG-NM_145979 for Chd4). TRC clone numbers are available upon request. Luciferase assays were performed using dual luciferase reporter assay system (Promega, E1910). MEF and HEK293 cells were transfected using Lipofectamine 2000 (Life Technologies). Human Mbd3 stealth siRNA were employed (Life Technologies, hss147580 and hss147581). The different constructs of human Netrin-1 promoter were already described^23^. HA-Flagged Netrin-1 cDNA was cloned into the *Pac*I and *Xho*I restriction sites of pcagg-ires-puro plasmid giving rise to pcagg-Netrin-1-ires-puro vector (pcagg-ires-puro is a kind gift from Ian Chambers). ES cells were stably transfected using FugeneHD reagent (Promega, E2691).

### Antibodies

Primary antibodies used in this study for immunofluorescence and FACS are as follows: anti-Oct4 (Santa Cruz, C10), anti-Nanog (Cosmobio, RCA B000 2P-F), anti-thy1 (Ebiosciences, 53-2.1), anti-SSEA1 (Stem Cell Technologies, 60060PE) and NL493-conjugated anti-SSEA4 antibody (R&D Systems, SC023), anti-Tra1-60 (Stemgent, 09-0009).

### Retroviral and lentiviral production

Plat-E cells were used to produce pMXs-based retroviruses containing the cDNA for Oct4, Sox2, Klf4 or cMyc, as previously described^32^. PlatE-generated mCherry retrovirus was used to monitor MEF infection efficiency in each experiment. 293T cells were used to produce lentiviral pLKO-derived particles.

### Mouse iPS cells generation

For iPS induction, MEFs were seeded per well of a six-well plate and 12 h later overnight infections were performed using equal amounts of each indicated retrovirus in the presence of 8 μg ml^−1^ polybrene (Sigma, 107689). The day after, wells were rinsed twice and cells were cultivated in medium supplemented or not with 150 or 750 ng ml^−1^ recombinant Netrin-1 (rNetrin-1). Forty-eight hours after infection, MEFs were reseeded onto irradiated feeders in iPS media. Media were replaced every day with medium alone or freshly supplemented with rNetrin-1. Emerging iPS colonies were monitored until days 12–14 when cells were harvested or individual colonies picked for further analysis. Similar protocol was employed with RNA sendai virus (cytotune 2.0, Life Technologies). The dox-inducible mice were purchased from the Jackson Laboratories (stock number 011001). MEFs, adult fibroblasts from ear and tail tip and intestinal epithelium were plated on irradiated feeders and treated with doxycycline at 2 μg ml^−1^. Recombinant Netrin-1 was purchased from Adipogen (AB40B-0075). Because of the poor stability of the recombinant Netrin-1 molecule and variability between batches, batch activity was systematically tested in cell death assays using UNC5b-transfected cells. The blocking Netrin-1 antibody was obtained from the Netris Pharma company.

### Human iPS cells generation

Reprogramming experiments have been performed on 5 × 10^5^ HFFs (Millipore) seeded in a well of six-well plates and grown in FibroGRO-LS medium (Millipore) until replating on MEFs. Cells were infected overnight with Sendaï viruses (Cytotune, Life Technologies) each of them at multiplicity of infection (MOI)=3. The day after, wells were rinsed twice and cells were cultivated in medium supplemented or not with 150 or 750 ng ml^−1^ rNetrin-1. Media were replaced every day with medium alone or freshly supplemented with rNetrin-1. Seven days post infection, cells were seeded onto freshly mitomycin-C-treated MEF at a density of 1 × 10^5^ or 2 × 10^5^ HFF cells per plate. The next day medium was switched to DMEM/F12 culture medium supplemented with 20% PluriQ Serum Replacement (GlobalStem), 0.1 mmol l^−1^ non-essential amino acids, 1 mmol l^−1^ L-glutamine, 0.1 mmol l^−1^ 2-mercaptoethanol, penicillin/streptomycin (all of them from Life Technologies), 12.5 ng ml^−1^ recombinant human basic fibroblast growth factor (Milytenyi Biotec). At day 26, fully reprogrammed colonies were manually picked and transferred to freshly mitomycin-C-treated MEF for amplification.

### Apoptosis detection

Cells were stained with anti-thy1 and apoptosis quantified with the Dead Cell Apoptosis Kit with Annexin V FITC and DAPI (Life technologies, V13242).

Colony formation assay was performed as previously described^32^.

### FACS sorting

Analysis was performed on a BD LSRFortessa. Sorting was performed on a BD FACSDiVa. Cells were sorted, washed immediately and centrifuged before being plated directly in fresh medium or frozen for RNA extraction and gene expression analysis.

### RNA-Sequencing

RNA quality was analysed using a Bioanalyser (Agilent). Libraries were constructed and sequenced on an illumina Hiseq 2000 by the MGX company. GEO record series GSE54107.

### Teratoma formation assay

5 × 10^6^ iPS cells were injected under the kidney capsules of 7-week-old severe combined immunodeficient (SCID) mice (CB17/SCID, Charles River). After 3 weeks, the mice were euthanized and lesions were surgically removed and fixed in formol or in 4% paraformaldehyde for cryosections. Same procedure was employed with 5 × 10^5^ iPS cells injection in testicles. Human teratoma formation was conducted as previously described^34^. All animal procedures were performed in accordance with the institutional guidelines (French ceccapp project 01369.01).

### Quantifications and Statistics

Comparison of the reprogramming efficiencies was performed on littermate embryos. At least two different batches of MEFs and reagents were used. Statistical analyses of mean and variance were performed with Prism 6 (GraphPad Software) and Student's *t*-test. The littermates belong to an experimental group determined by the genotyping.

## Additional information

**How to cite this article:** Ozmadenci, D. *et al.* Netrin-1 regulates somatic cell reprogramming and pluripotency maintenance. *Nat. Commun.* 6:7398 doi: 10.1038/ncomms8398 (2015).

## Supplementary Material

Supplementary Information Supplementary Figures 1-4

## References

[b1] Takahashi K. & Yamanaka S. Induction of pluripotent stem cells from mouse embryonic and adult fibroblast cultures by defined factors. Cell 126, 663–676 (2006). 16904174 10.1016/j.cell.2006.07.024

[b2] Takahashi K. *et al.* Induction of pluripotent stem cells from adult human fibroblasts by defined factors. Cell 131, 861–872 (2007). 18035408 10.1016/j.cell.2007.11.019

[b3] Lavial F. *et al.* The Oct4 homologue PouV and Nanog regulate pluripotency in chicken embryonic stem cells. Development 134, 3549–3563 (2007). 17827181 10.1242/dev.006569

[b4] Kawamura T. *et al.* Linking the p53 tumour suppressor pathway to somatic cell reprogramming. Nature 460, 1140–1144 (2009). 19668186 10.1038/nature08311 PMC2735889

[b5] Li Y. *et al.* The p53-PUMA axis suppresses iPSC generation. Nat. Commun. 4, 2174 (2013). 23873265 10.1038/ncomms3174 PMC4394110

[b6] Marion R. M. *et al.* A p53-mediated DNA damage response limits reprogramming to ensure iPS cell genomic integrity. Nature 460, 1149–1153 (2009). 19668189 10.1038/nature08287 PMC3624089

[b7] Evan G. Cancer. Taking a back door to target Myc. Science 335, 293–294 (2012). 22267799 10.1126/science.1217819

[b8] Mehlen P., Delloye-Bourgeois C. & Chedotal A. Novel roles for Slits and netrins: axon guidance cues as anticancer targets? Nat. Rev. Cancer. 11, 188–197 (2011). 21326323 10.1038/nrc3005

[b9] Cirulli V. & Yebra M. Netrins: beyond the brain. Nat. Rev. Mol. Cell Biol. 8, 296–306 (2007). 17356579 10.1038/nrm2142

[b10] Mazelin L. *et al.* Netrin-1 controls colorectal tumorigenesis by regulating apoptosis. Nature 431, 80–84 (2004). 15343335 10.1038/nature02788

[b11] Castets M. *et al.* Inhibition of endothelial cell apoptosis by netrin-1 during angiogenesis. Dev. Cell 16, 614–620 (2009). 19386270 10.1016/j.devcel.2009.02.006

[b12] Soufi A., Donahue G. & Zaret K. S. Facilitators and impediments of the pluripotency reprogramming factors' initial engagement with the genome. Cell 151, 994–1004 (2012). 23159369 10.1016/j.cell.2012.09.045 PMC3508134

[b13] Mikkelsen T. S. *et al.* Dissecting direct reprogramming through integrative genomic analysis. Nature 454, 49–55 (2008). 18509334 10.1038/nature07056 PMC2754827

[b14] Wernig M. *et al.* A drug-inducible transgenic system for direct reprogramming of multiple somatic cell types. Nature Biotechnol. 26, 916–924 (2008). 18594521 10.1038/nbt1483 PMC2654269

[b15] Polo J. M. *et al.* A molecular roadmap of reprogramming somatic cells into iPS cells. Cell 151, 1617–1632 (2012). 23260147 10.1016/j.cell.2012.11.039 PMC3608203

[b16] Bernet A. *et al.* Inactivation of the UNC5C Netrin-1 receptor is associated with tumor progression in colorectal malignancies. Gastroenterology 133, 1840–1848 (2007). 17967459 10.1053/j.gastro.2007.08.009 PMC2211510

[b17] Thibert C. *et al.* Inhibition of neuroepithelial patched-induced apoptosis by sonic hedgehog. Science 301, 843–846 (2003). 12907805 10.1126/science.1085405

[b18] Mehlen P. *et al.* The DCC gene product induces apoptosis by a mechanism requiring receptor proteolysis. Nature 395, 801–804 (1998). 9796814 10.1038/27441

[b19] Castets M. *et al.* DCC constrains tumour progression via its dependence receptor activity. Nature 482, 534–537 (2012). 22158121 10.1038/nature10708

[b20] Buganim Y. *et al.* Single-cell expression analyses during cellular reprogramming reveal an early stochastic and a late hierarchic phase. Cell 150, 1209–1222 (2012). 22980981 10.1016/j.cell.2012.08.023 PMC3457656

[b21] Stadtfeld M., Maherali N., Breault D. T. & Hochedlinger K. Defining molecular cornerstones during fibroblast to iPS cell reprogramming in mouse. Cell Stem Cell 2, 230–240 (2008). 18371448 10.1016/j.stem.2008.02.001 PMC3538379

[b22] Chen X. *et al.* Integration of external signaling pathways with the core transcriptional network in embryonic stem cells. Cell 133, 1106–1117 (2008). 18555785 10.1016/j.cell.2008.04.043

[b23] Delloye-Bourgeois C. *et al.* Nucleolar localization of a netrin-1 isoform enhances tumor cell proliferation. Sci. Signal. 5, ra57 (2012). 10.1126/scisignal.2002456 22871610

[b24] Alder O. *et al.* Ring1B and Suv39h1 delineate distinct chromatin states at bivalent genes during early mouse lineage commitment. Development 137, 2483–2492 (2010). 20573702 10.1242/dev.048363 PMC2927698

[b25] Ang Y. S. *et al.* Wdr5 mediates self-renewal and reprogramming via the embryonic stem cell core transcriptional network. Cell 145, 183–197 (2011). 21477851 10.1016/j.cell.2011.03.003 PMC3097468

[b26] Rais Y. *et al.* Deterministic direct reprogramming of somatic cells to pluripotency. Nature 502, 65–70 (2013). 24048479 10.1038/nature12587

[b27] dos Santos R. L. *et al.* MBD3/NuRD facilitates induction of pluripotency in a context-dependent manner. Cell Stem Cell 15, 102–110 (2014). 24835571 10.1016/j.stem.2014.04.019 PMC4082719

[b28] Tang F. *et al.* Deterministic and stochastic allele specific gene expression in single mouse blastomeres. PLoS ONE 6, e21208 (2011). 21731673 10.1371/journal.pone.0021208 PMC3121735

[b29] Krimpenfort P. *et al.* Deleted in colorectal carcinoma suppresses metastasis in p53-deficient mammary tumours. Nature 482, 538–541 (2012). 22358843 10.1038/nature10790

[b30] Delloye-Bourgeois C. *et al.* Interference with netrin-1 and tumor cell death in non-small cell lung cancer. J. Natl Cancer Inst. 101, 237–247 (2009). 19211441 10.1093/jnci/djn491

[b31] Furne C., Rama N., Corset V., Chedotal A. & Mehlen P. Netrin-1 is a survival factor during commissural neuron navigation. Proc. Natl Acad. Sci. USA 105, 14465–14470 (2008). 18796601 10.1073/pnas.0803645105 PMC2567197

[b32] Percharde M. *et al.* Ncoa3 functions as an essential Esrrb coactivator to sustain embryonic stem cell self-renewal and reprogramming. Genes Dev. 26, 2286–2298 (2012). 23019124 10.1101/gad.195545.112 PMC3475801

[b33] Lavial F. *et al.* Bmi1 facilitates primitive endoderm formation by stabilizing Gata6 during early mouse development. Genes Dev. 26, 1445–1458 (2012). 22713603 10.1101/gad.188193.112 PMC3403013

[b34] Griscelli F. *et al.* Malignant germ cell-like tumors, expressing Ki-1 antigen (CD30), are revealed during *in vivo* differentiation of partially reprogrammed human-induced pluripotent stem cells. Am. J. Pathol. 180, 2084–2096 (2012). 22425713 10.1016/j.ajpath.2012.01.011

