# Supplementary material for: Netrin-1 regulates somatic cell reprogramming and pluripotency maintenance
Source: Nat Commun. 2015 Jul 8;6:7398. doi: 10.1038/ncomms8398 (PMC4510695; doi:10.1038/ncomms8398)
Supplement: Supplementary Information — Supplementary Figures 1-4 [file ncomms8398-s1.pdf]

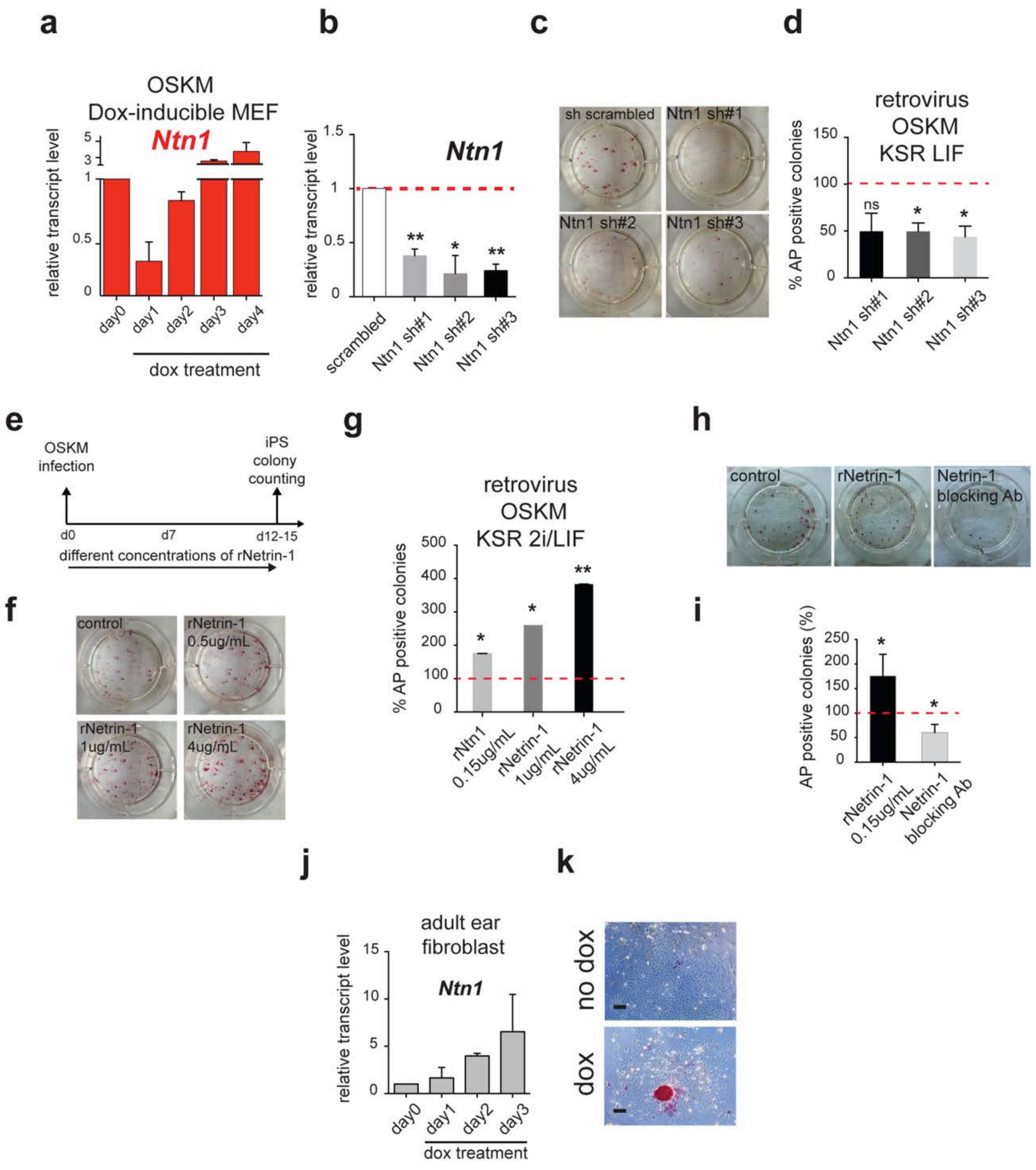

Supplementary Figure 1. Netrin-1 controls reprogramming.

(a) *Ntn1* expression is biphasic during OSKM dox-inducible MEF reprograming. Q-RTPCR depicts *Ntn1* expression during reprogramming induced by dox treatment. Data are normalized to housekeeping genes and expressed relative to MEF as the mean ± s.d. (n=2). (b) Knockdown efficiency of Ntn1 shRNAs. Q-RTPCR depicts *Ntn1* expression level 96 hours following MEF infection with lentiviral particles encoding 3 different shRNA targeting *Ntn1*. Ntn1 expression level in sh scrambled MEF is set at 1. Data are the mean ± s.d. (n=2). (c-d) Decreased reprogramming efficiency following Netrin-1 depletion, analysed by quantifying alkaline phosphatase positive iPS colonies. The number of colonies produced from sh-scrambled infected MEFs is set at 100% for each individual experiment. Data are the mean ± s.d. (n=3). Experiments were performed with different batches of MEFs and viruses. Statistical analysis, Student's t-test p<0,05 (*), ns: non significant. (e-g)

Recombinant Netrin-1 impact on mouse iPS cells generation induced by OSKM retroviral infection. (e) Schematic of the time schedule of recombinant Netrin-1 treatment experiment. (f-g) rNetrin-1 (0.15µg/mL, 1 or 4µg/mL) was added daily to the culture media and AP positive colonies counted 12-14d following OSKM infection. The number of colonies produced from untreated MEFs is set at 100% for each individual experiment. Data are the mean ± s.d. (n=3). Statistical analysis, Student's t-test p<0,05 (*) or p<0,01 (**). (h-i) Recombinant Netrin-1 and Netrin-1 blocking antibody impact on reprogramming efficiency. Netrin-1 (0.15µg/mL) and Netrin-1 blocking antibody (10µg/mL) were added daily to the culture media and AP+ colonies counted 12-14d following OSKM infection. Data are the mean ± s.d. (n=3). Statistical analysis, Student's t-test p<0,05 (*). (j) Netrin-1 expression profile during reprogramming of adult ear fibroblasts. Q-RTPCR depicts *Ntn1* expression during a 3 days kinetic of dox treatment. Data are the mean ± s.d. of two independent experiments. (k) Mouse intestinal epithelium reprogramming. Intestinal epithelium was dissociated and plated in culture on irradiated feeder. After 15 days in absence (upper panel) or in presence (lower panel) of dox, alkaline phosphatase staining was performed. Bars 200µm.

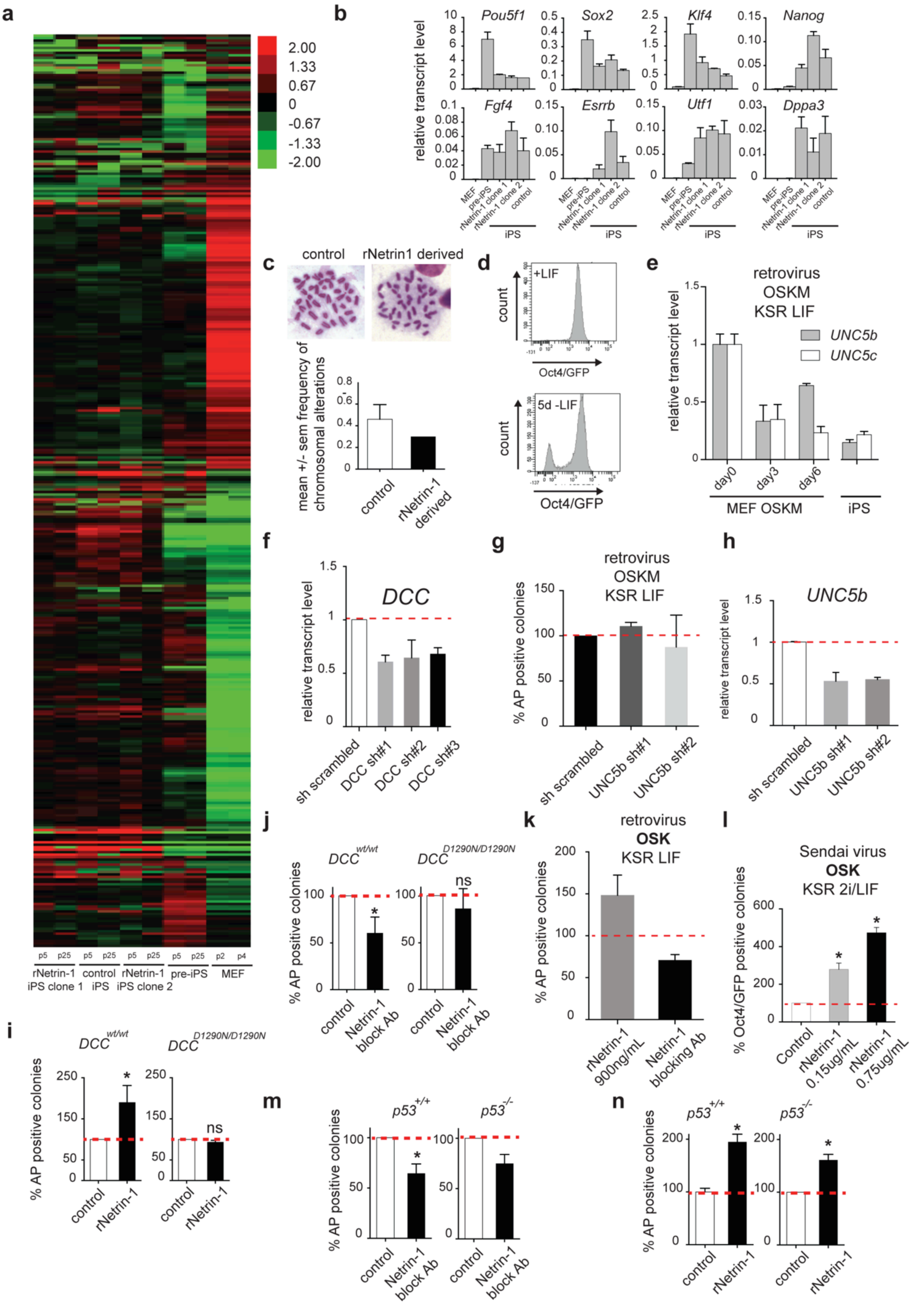
a
2.00
1.33
0.67
0
-0.67
-1.33
-2.00
p5 p25 p5 p25 p5 p25 p5 p25 p2 p4
rNetrin-1 iPS clone 1
control iPS
rNetrin-1 iPS clone 2
pre-iPS
MEF
b
relative transcript level
Pou5f1
Sox2
Klf4
Nanog
Fgf4
Esrrb
Utf1
Dppa3
MEF
pre-iPS
rNetrin-1 clone 1
rNetrin-1 clone 2
control
iPS
c
control
rNetrin1 derived
mean +/- sem frequency of chromosomal alterations
control
rNetrin-1 derived
d
+LIF
count
Oct4/GFP
5d -LIF
e
retrovirus
OSKM
KSR LIF
relative transcript level
UNC5b
UNC5c
day0
day3
day6
MEF OSKM
iPS
f
DCC
relative transcript level
sh scrambled
DCC sh#1
DCC sh#2
DCC sh#3
g
retrovirus
OSKM
KSR LIF
% AP positive colonies
sh scrambled
UNC5b sh#1
UNC5b sh#2
h
UNC5b
relative transcript level
sh scrambled
UNC5b sh#1
UNC5b sh#2
i
DCC wt/wt
DCC D1290N/D1290N
% AP positive colonies
control
rNetrin-1
ns
j
DCC wt/wt
DCC D1290N/D1290N
% AP positive colonies
control
Netrin-1 block Ab
ns
k
retrovirus
OSK
KSR LIF
% AP positive colonies
rNetrin-1 900ng/mL
Netrin-1 blocking Ab
l
Sendai virus
OSK
KSR 2i/LIF
% Oct4/GFP positive colonies
Control
rNetrin-1 0.15ug/mL
rNetrin-1 0.75ug/mL
m
p53 +/+
p53 -/-
% AP positive colonies
control
Netrin-1 block Ab
n
p53 +/+
p53 -/-
% AP positive colonies
control
rNetrin-1

Supplementary Figure 2. Netrin-1 is not detrimental to mouse and human iPS cell quality.

(a) Dendogram of RNA-sequencing data from mouse Oct4/GFP MEF, pre-iPS, “control” and “rNetrin-1 derived” iPS cells obtained following OSKM retroviral infection. (b) "Control" and "rNetrin-1 derived" mIPS cells are successful at turning on naive pluripotency markers. RNA-Seq data for *Pou5f1*, *Sox2*, *Klf4*, *Nanog*, *Fgf4*, *Esrrb*, *Utf1* and *Dppa3* expression levels in MEF, pre-iPS, control and rNetrin-1 derived iPS cells. Data are normalized to housekeeping genes as the mean ± s.d. (n=3). (c) Chromosomes counting in “control" and “rNetrin-1 derived” mouse iPS cells. (d) Mouse “rNetrin-1 derived” iPS cells are able to downregulate endogenous Oct4 promoter upon LIF withdrawal. Cells were induced to differentiate for 5 days by LIF withdrawal and subjected to FACS analysis for Oct4/GFP expression. (e) *UNC5b* and *UNC5c* dynamics during pluripotent reprogramming. Q-RTPCR depicts *UNC5b* and *UNC5c* expression levels during a 6 days kinetic following OSKM retroviral infection. Data are normalized to housekeeping genes and expressed relative to MEF as the mean ± s.d. (n=3). (f) Knockdown efficiency of DCC shRNA. Q-RTPCR depicts *DCC* expression level 96 hours following MEF infection with lentiviral particles encoding 3 different shRNA against DCC. Data are the mean ± s.d. (n=2). (g) Effect of *UNC5b* depletion on reprogramming efficiency induced by OSKM retroviral infection, analysed by quantifying alkaline phosphatase (AP) positive colonies. The number of colonies produced from sh-scrambled-infected MEFs is set at 100% for each individual experiment. Three different shRNA were used. Data are the mean ± s.d. (n=3). (h) Knockdown efficiency of UNC5b shRNA. Q-RTPCR depicts *UNC5b* expression level 96 hours following NIH3T3 infection with lentiviral particles encoding 2 different shRNA against UNC5b. Data are the mean ± s.d. (n=2). (i-j) Netrin-1 effect on reprogramming is due to DCC pro-apoptotic activity. Recombinant Netrin-1 and Netrin-1 blocking antibody treatments were performed in parallel in $DCC^{wt/wt}$ and $DCC^{D1290N/D1290N}$ MEF. The number of AP positive colonies produced from untreated MEF is set at 100% for each individual experiment. Data are the mean ± s.d. (n=3). Statistical analysis, Student's t-test p<0,05 (*), ns: non significant. (k-n) Netrin-1 effect on reprogramming is mainly c-Myc and p53 independent. (k) Recombinant Netrin-1 and Netrin-1 blocking antibody impact on reprogramming induced by OSK retroviral infection. Recombinant Netrin-1 (0.9μg/mL) and Netrin-1 blocking antibody (10μg/mL) were added daily to the culture media and AP+ colonies counted 20-25 days following OSKM infection. The number of colonies produced from untreated MEFs is set at 100% for each individual experiment. Data are the mean ± s.d. (n=3). (l) Recombinant Netrin-1 impact on mouse iPS cells generation induced by OSK RNA sendai virus. Netrin-1 (0.15μg/mL or 0.75μg/mL) was added daily to the culture media and Oct4/GFP positive colonies counted 20-25 days following OSK infection. The number of colonies produced from untreated MEFs is set at 100% for each individual experiment. Data are the mean ± s.d. (n=3). Statistical analysis, Student's t-test p<0,05 (*). (m-n) Recombinant Netrin-1 and Netrin-1 blocking antibody effect on *p53-/-* MEF reprogramming. Treatments were performed in parallel in $p53^{+/+}$ and $p53^{-/-}$ MEF. The number of AP positive colonies produced from untreated MEF is set at 100% for each individual experiment. Same results are obtained with at least 2 different MEF clones for each genotype. Transduction efficiencies were similar between genotype. Statistical analysis, Student's t-test p<0,05 (*).

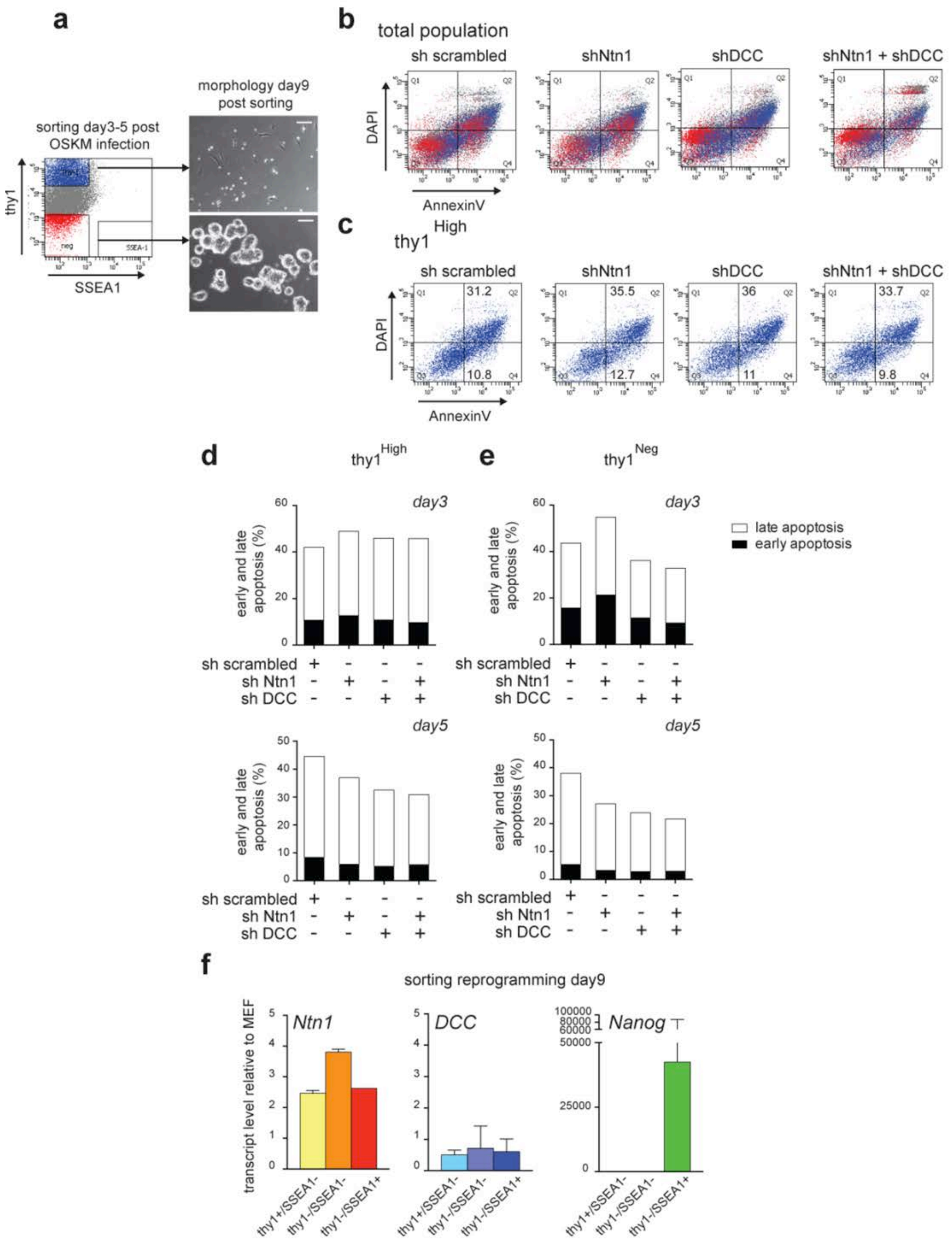

Supplementary Figure 3. Netrin-1/DCC balance modulates apoptosis during the early phase of reprogramming.

(a) Representative FACS profile of thy1^{High} and thy1^{Neg} cells 3-5 days post OSKM infection. Cells were sorted and plated back in reprogramming media for 6-9 additional days before brightfield pictures were taken. Bars 150µm. (b-c) Representative FACS profiles of apoptosis analysis in bulk population (b) or in thy1^{High} cells (c) following Ntn1 and/or DCC depletion. Cells were immunostained with thy1, AnnexinV and DAPI after 3 and 5 days of pluripotent reprogramming. (d-e) Quantification of early and late apoptotic cells percentage in thy1^{High} and thy1^{Neg} subpopulations following Ntn1 and/or DCC depletion. Values correspond to one experiment representative of three independent experiments. (f) *Ntn1*, *DCC* and *Nanog* expression levels in subpopulations of cells during reprogramming. Cells were sorted on thy1 and SSEA1 cell surface markers at day9 post-OSKM infection and Q-RTPCR

performed. Data are normalized to housekeeping genes and expressed relative to MEF as the mean ± s.d. (n=3).

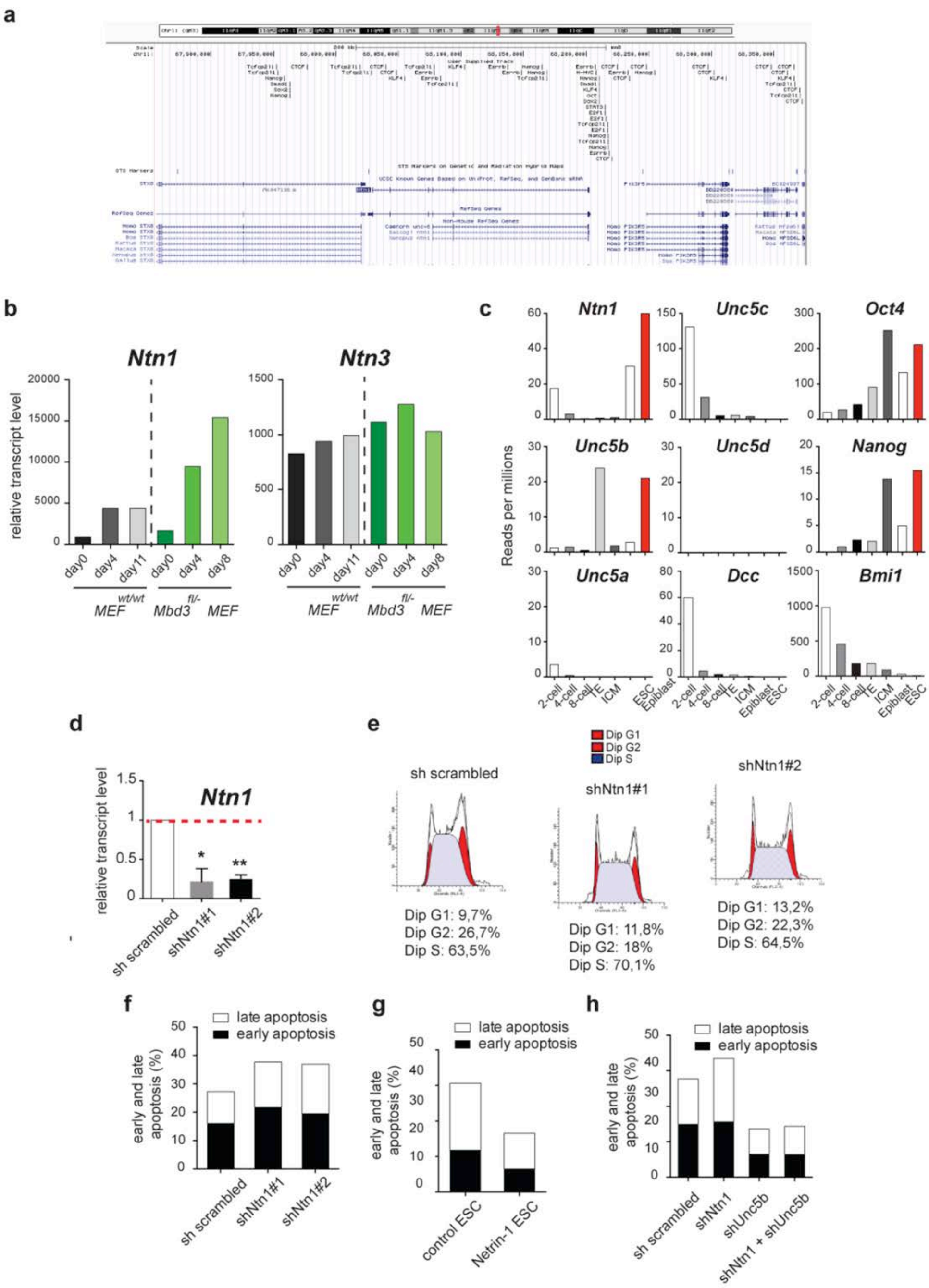

Supplementary Figure 4. Netrin-1 transient transcriptional repression is mediated by the NuRD complex.

(a) Pluripotency factor binding on Netrin-1 promoter in mouse ES cells. Data are extracted from[22]. (b) *Ntn1* expression level during *MEF*$^{wt/wt}$ and *Mbd3*$^{fl/-}$ reprogramming. Data are extracted from published resources[26]. (c) *Ntn1*, *UNC5a-d*, *DCC*, *Oct4*, *Nanog* and *Bmi1* expression at the single cell level in pre-implantating embryos, epiblast and ES cells. Reads per millions (RPM) for each gene was extracted from[28]. (d) Netrin-1 knockdown in stable ES cell lines. Q-RTPCR depicts *Ntn1* expression in sh scrambled, shNtn1#1 and shNtn1#2 stable ES cell lines obtained by lentiviral infection. Data are normalized to housekeeping genes and expressed relative to sh scrambled ES cells as the mean ± s.d. (n=3). (e) Netrin-1 depletion in ES cells has no effect on cell cycle distribution. Cell cycle was

analysed by FACS using PI using the FACS calibur software. (f) Quantification of early and late apoptotic cells percentage in sh scrambled, shNtn1#1 and shNtn1#2 ES cells. Values correspond to one experiment representative of three independent experiments. (g) Quantification of early and late apoptotic cells percentage in "control" and "Netrin-1 expressing" ES cells. Values correspond to one experiment representative of three independent experiments. (h) Quantification of early and late apoptotic cells percentage upon Ntn1 and/or UNC5b depletion. Values correspond to one experiment representative of two independent experiments.
